# Wireless Sensors Grouping Proofs for Medical Care and Ambient Assisted-Living Deployment

**DOI:** 10.3390/s16010033

**Published:** 2016-01-02

**Authors:** Denis Trček

**Affiliations:** Faculty of Computer and Information Science, University of Ljubljana, Večna pot 113, Ljubljana 1000, Slovenia; denis.trcek@fri.uni-lj.si; Tel.: +386-1-4768-918; Fax: +386-1-4264-647

**Keywords:** wireless networks, internet of things, health care, ambient assisted living, PPDR networks, RFID, lightweight protocols, security, Yoking proofs

## Abstract

Internet of Things (IoT) devices are rapidly penetrating e-health and assisted living domains, and an increasing proportion among them goes on the account of computationally-weak devices, where security and privacy provisioning alone are demanding tasks, not to mention grouping proofs. This paper, therefore, gives an extensive analysis of such proofs and states lessons learnt to avoid possible pitfalls in future designs. It sticks with prudent engineering techniques in this field and deploys in a novel way the so called non-deterministic principle to provide not only grouping proofs, but (among other) also privacy. The developed solution is analyzed by means of a tangible metric and it is shown to be lightweight, and formally for security.

## 1. Introduction

Rapid proliferation of pervasive computing objects, like sensors and RFIDs, significantly changes not only the applications landscape, but also increases concerns related to security, privacy and even safety and this especially holds true in medical settings. The population of this kind of devices is quite heterogeneous. Despite this diversity, a notable common property is limited power supply, while a large part of them also lacks computing resources. A straightforward consequence is a requirement for lightweight cryptographic protocols. Knowing that cryptographic protocols design, alone, is a tricky issue, adding stringent computing resources and power limitations results in additional complexity. Knowing further that these devices are becoming rapidly adopted in medical settings, appropriate solutions are of utmost importance (for those interested, an extended survey that covers IoT security and energy issues can be found in [1]).

**Figure 1:**
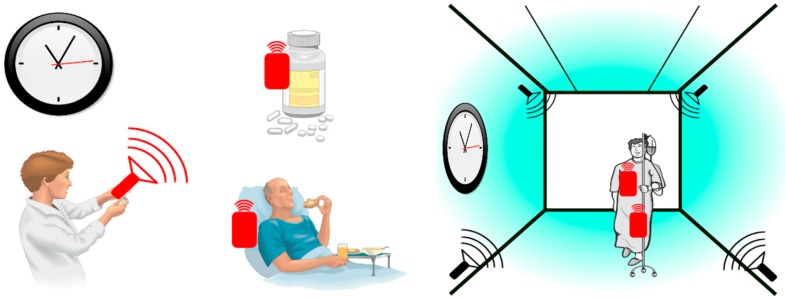
Two application scenarios for Yoking proofs (drugs administration and ubiquitous patients’ physiological functions monitoring in clinics or ambient assisted living).

This research paper addresses an important, frequently considered problem in medical settings where a cluster of computationally-weak devices (e.g, RFIDs) has to act in orchestration. Such clusters should act in a way where it can be proved that the responses are obtained simultaneously. Traditional application areas are drugs administration, but clinical experiments are taking place for tracing patient’s physiological signals from a wireless body area networks while she moves around hospital’s premises as shown in Figure 1 (an early such can be found in [2], while more recent on in [3]). It should be added that the application areas are by no means limited to medical domain—they range from industrial assembly lines to logistics and PPDR (public protection and disaster relief) settings.

The strategy in this paper is to incrementally build upon protocols that have already been subject to scientific scrutiny. The reasoning is as follows: knowing that design of a watertight cryptographic protocol can be an elusive endeavor, it makes sense to avoid designing a new solution from scratch to minimize the possibility that the developed solution would turn out to be just another Pandora’s Box [4]. By doing so this paper exposes important issues that seem to be often overlooked recently and which were already stated by Abadi and Needham in the 1990s—these are prudent engineering principles [5]. Last but not least, formal verification is a must for proving authentication and confidentiality (privacy) properties of developed Yoking proof schemes, which is the case with the developed solution in this paper.

The paper is structured as follows. There is an overview of the field in the second section. In the third section it is followed by an analysis of solutions that present the basis for new protocols, which are given in the same section. In the fourth section these are analyzed from two perspectives: authentication and privacy provisioning and lightweight properties perspective. There are conclusions in the fifth section, while the paper ends up with acknowledgements, references, appendix and author’s *vita*.

## 2. Overview of the Field

Proofs of simultaneous presence of RFIDs were introduced by Juels in 2004, when he coined the term Yoking proofs [6]. These are intended to cover scenarios where proofs of simultaneous scanning of RFIDs are needed. For their analysis and design presented in this paper the following notation will be used:

- A, B, C, and X denote entities (tags’ identifiers), V denotes a verifier and R denotes a reader;
- *r* denotes a random value (also a nonce), while its subscript denotes an entity that has generated it (e.g., *r*_A_ denotes a random value generated by A);
- *s*_A,B_ and *x*_A,B_ denote a secret key *x* or secret *s* that is shared between entities A and B, while *x*_A_ and *x*_B_ denote A’s and B’s own secrets (known also only to a server);
- MAC*_s_*[*m*]/MAC*_x_*[*m*] denotes a cryptographic (hashed) message authentication code obtained by using a secret key *x* or *s* that is , e.g., appended to a message *m* before hashing;
- P_AB_ denotes a proof that entities (objects) A and B were scanned simultaneously.

**Figure 2:**
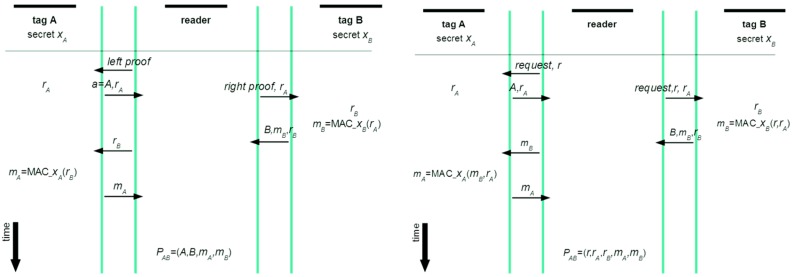
Juels’ Yoking proof protocol (**Left**), and Piramuthu’s extension of Juels’ protocol (**Right**).

Now starting with the first solutions proposed by Juels—their advantage was clear and minimalistic design, which especially holds true for the lightweight variant that is based on MACs—it is given in Figure 2. This work has gained interest and soon research followed that found attacks against it. The first one was done by Saito and Sakurai where they proposed timestamps to cure the discovered weakness [7]. However, this improved scheme was also vulnerable to reply attacks, as discovered by Piramuthu [8]. If an attacker submits a future timestamp and obtains the response from the first tag he can use it later on when the corresponding time becomes true. Then the attacker queries the second tag and completes the protocol ending up with a proof that both tags have been read simultaneously, although this has not been the case.

Therefore Piramuthu has proposed an improvement that is given in Figure 2. According to Piramuthu, this solution should also provide privacy.

**Figure 3:**
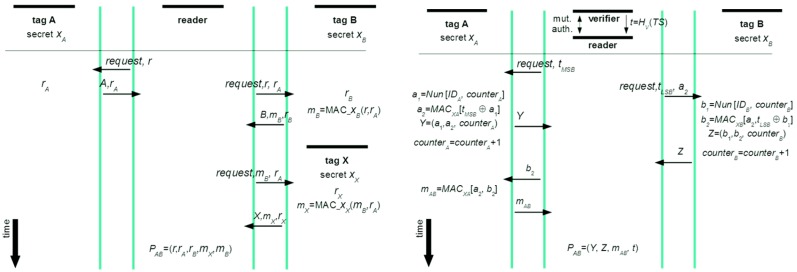
An attack on Piramuthu’s protocol by Peris-Lopez *et al.* (**Left**) and Clumping proof scheme (**Right**).

There are many unclear issues with Piramuthu’s version. It is not clear how values for *r_A_* and *r_B_* are produced. If *r* from a reader serves as an input, then being interrogated by the same “*r*” tags will produce the same *r*_A_, which gives good grounds to attacks because of not adhering to prudent security protocol engineering principles [5]. Next, how can a prover find A’s and B’s identities on the basis of received (*r*, *r_A_*, *r_B_*, *m_X_*, *m_B_*)? Further, Piamuthu’s improvement is claimed to be vulnerable and the attack is shown in Figure 2 [9]. However, this procedure is actually not revealing a weakness in Piramuthus’s protocol. If following exactly the attack as described, this attack cannot be successful. Actually, *P_AB_* has to be verifiable in its entirety. Thus, a verifier can find out that the key for verification of *m_X_* differs from that of A. If authors wanted to claim that the protocol provides proof *P_XB_*, then also this claim would be problematic. There can be a time delay between interrogation of B and *X*, but the verification of will still be fine. However, authors in [9] that there is no privacy identifiers of tags A and B are sent in plaintext. Further, they present a new scheme called Clumping proof, where they include, explicitly, the verifier in the proof-generating phase. This entity is first mutually authenticated with a reader, and afterward a timestamp *TS* is exchanged. As opposed to Piramuthu’s scheme, this timestamp is encrypted by the verifier. This encrypted value *t* of timestamp, which is obtained by a keyed hash function *H* (*i.e.*, *t* = *H*_V_(*TS*)), is divided into its upper part (MSB) and its lower part (LSB)—the MSB part is exchanged with tag A and the LSB part with tag B. Further, changeable identifiers are introduced that act as pseudonyms (a special kind of function called Nun is used for this purpose). Further, counters in tags are introduced to make explicit to which run of a protocol particular messages belong to—the whole scheme is presented in Figure 3.

The Clumping proof scheme does introduce some improvements, one of them being use of counters and the other one being (almost strict) chaining of all calculations. Nevertheless, this scheme needs an improvement—the hashed timestamp is an inherent point of attack. An attacker can shuffle a bit (or some bits) of the timestamp exchanged at the beginning between the verifier and the reader, and this cannot be noticed until the verifier checks the proof. To cure the situation, the verifier and the reader should share a common secret and use it to obtain a hashed message integrity code that is appended to the exchanged hashed timestamp. This timestamp has to be verified by the reader before the hashed LSB and MSB parts are transmitted to tags. By the same token ideally, tags should perform such integrity checks as well.

Extending further the above line of reasoning leads to very interesting consequences that have not been discussed in the literature so far. Despite the above-described attack, and assuming that the subsequent exchanges of messages are not attacked, the calculations following the attacked time stamp exchange are still providing a usable output. This output does not provide a proof that the tags were scanned synchronously at a certain absolute time, but a proof that the tags were scanned simultaneously at some (undefined) temporal point. Further, from the tags’ point of view timestamps are semantically equivalent to nonces and random values. While freshness of nonces and random values can be verified to some extent by RFIDs (see, e.g., [4]), the verification of timestamps (*i.e.*, absolute time values) should not be assumed because such checks are very demanding for RFIDs. Adding a fact that authentication of readers by tags is also very demanding, the provisioning of grouping proofs with exact time is dependent on (honest) readers. Interestingly, authors require an on-line involvement of a verifier and its interaction with a reader. As a result, one actually ends up with a variant of on-line (authentication) protocol.

Lessons learned so far remain valid for research that followed the above described designs. In Cho *et al*., presented a variant of Piramuthu’s protocol [10]. Later, protocols that support anonymization appeared including Burmester *et al.* [11] and Chien and Liu [12]. In this latter paper authors state that malfunctioning or malicious tags can lead to calculation of useless proofs and consequently, denial of service attacks. Therefore on-line authentication schemes should be deployed and they propose one. However, the same authors later developed another off-line grouping verification scheme [13]. Probably the reason is that on-line grouping proofs are better replaced by on-line authentication schemes where proofs of simultaneous reading are formed by trusted back-ends.

All these protocols are analyzed for their weaknesses and computational costs in [14] (interestingly, Yoking proofs are all but simple even the recent proposal by [15] has been found vulnerable [16]). On this basis guidelines for attacks resistant grouping protocols design are given that can be summarized as follows. The basic design issue is severely restricted power supply (so tags should compute just, e.g., pseudo-random message authentication codes). Next, except for the first one, every input to a given tag should be a derivation of computations that can be only carried out by fellow tags participating in the proof. Next, if possible, tags should use group identifiers and group keys to prove membership to a group and should check each other’s computations to make sure that only group members participate in the proof. Next, time stamps should be used to thwart replay attacks. Next, performance issues should be considered (and some approximate metrics is provided for this purpose). Next, forward secrecy should be enabled, so when secret keys are disclosed all dependent past cryptographic calculations should not be endangered. Finally, privacy is of a prime concern, therefore dynamic anonymous identifiers have to be used.

Taking into account what has been stated so far the above guidelines have to be adjusted as follows. Grouping proofs can be off-line or on-line. With off-line proofs a reader is not able to verify the calculations, thus there is an inherent risk of producing false proofs (be it on the account of attacking tags, or attackers that interfere with exchanged messages, *etc.*), because such cases can be detected only during the verification phase. Next, use of group IDs and group keys is desired, but it limits applicability of grouping proofs, so such identifiers should not be generally assumed. Next, checks of previous calculations by fellow tags are desired, but due to severely restrained resources they should not be generally assumed (this requirement goes against the famous 5 cents production costs limit). Next, as timestamps have the same semantics to RFIDs as random values and nonces, their use adds security only in case of a trusted reader. Next, performance metrics should be as fine graded as possible. One such metric that nails down the protocol’s requirements to the number of NAND gates, which reflects technological reality, while at the same time it is transformable to power/energy consumption, is described in [4]. Finally, when designing privacy protocols, such ones should be the basis that have been subject to scientific scrutiny.

## 3. New Solution for Orchestrated Security and Privacy

**Figure 4:**
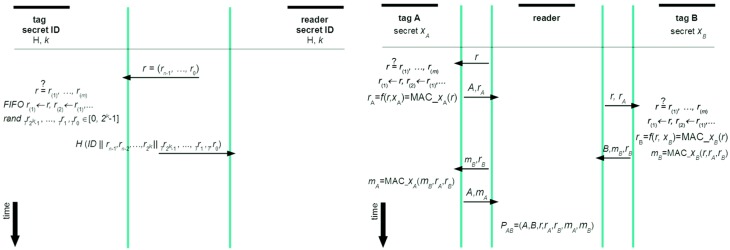
The ND-PEPS protocol (**Left**) and enhanced version of Juels/Piramuthu “Yoking proof” protocol (**Right**).

A seminal work in formal analysis of strength of privacy providing RFID protocols is given in [17] and among the strongest protocols in this domain is the protocol developed by Okhubo, Suzuki, and Kinoshita [18]. With this protocol this holds true for privacy provisioning, but its weakness is a practical deployment issue, because the protocol can be forced into de-synchronization with a database [4]. To prevent this kind of attack, and to preserve the strengths of Okhubo, Suzuki, and Kinoshita protocol, the non-deterministic approach, called ND-PEPS, will be used (its newer version that enables not only privacy, but also confidential exchange of, e.g., measured physiological quantities [4]). ND-PEPS is called non-deterministic because it deploys asymmetry in computational power between a tag and a reader (or verifier). More precisely, the tag computes randomized responses from a certain interval of possible responses (which is not computationally demanding), while the reader (which is verifier in our case) has to compute all possible responses and search for a match to identify the tag (which is computationally not hard for the verifier). The ND-PEPS protocol is shown in Figure 4 (a Dolev-Yao adversary model is assumed, tamper resistance of tags is assumed, and only the back-end server is trusted).

Now let a reader and a tag share a secret value *ID*, and also agree upon parameter *k* and a strong one-way hash function *H*. To authenticate the tag the reader sends to the tag a random query *r*. Upon receipt the tag compares the received *r* against previously received ones to prevent attacks with messages, which are not fresh. If this challenge is fresh, it is written in FIFO (first in, first out) memory and the oldest one is discarded. Afterward, the tag generates a random value *Tr*, replaces the last bits in the challenge with this value, and appends the result to the secret *ID*. This concatenated sequence is hashed and the output is sent to the reader. Upon receipt, the reader (*i.e.*, verifier) generates all possible values from the closed interval [0, 2^*k*−1^] for tags for which it keeps track in its database and obtains possible hashed sequences. Using these sequences and the received message the reader looks for a match—once it is found, the tag is authenticated.

### 3.1. Design of an Improved Grouping Proof Architecture

The basis for the solutions presented here is Juels’ protocol, which despite its deficiencies has many nice properties. It has a clean design and semantics of its steps, it sticks with a simplistic architecture approach and it is lightweight. Likely because of these properties it was a good candidate for successful analysis and discovery of weaknesses by Piramuthu, who proposed its improvements (as discussed above and shown in Figure 2). This architecture will be taken as a basis for improved version that provides also privacy through anonymization. Further, a seminal work on prudent engineering principles for designing cryptographic protocols [5] will be taken into account to remedy accordingly Piramuthu’s protocol weak points that could open doors to attacks.

Starting with the challenge *r* that is sent in the first step of the protocol run, its value makes no sense if not used by tag A. However it should play a crucial role as a value around which all messages are ‘chained’. Further, value *r*_A_ should depend on the challenge *r* and secret *x*_A_—the same chaining logic holds true for value *r*_B_, *m*_B_, and *m*_A_. Such structure of messages supports consistent semantic interpretation, while their logical linking provides a provably linked sequence that belongs to one particular run of the protocol (the whole protocol is given in Figure 4). Each tag checks the freshness of *r* (of course, to a limited extent) by comparing it with a sequence of previous challenges stored in a first-in-first-out (FIFO) memory. Note that the above architecture is a conceptual basis that serves for clarification of the derivation of the final (optimized and secure) solution, which will be presented in the next subsection.

### 3.2. A New Grouping Proof with Privacy Provisioning

**Figure 5:**
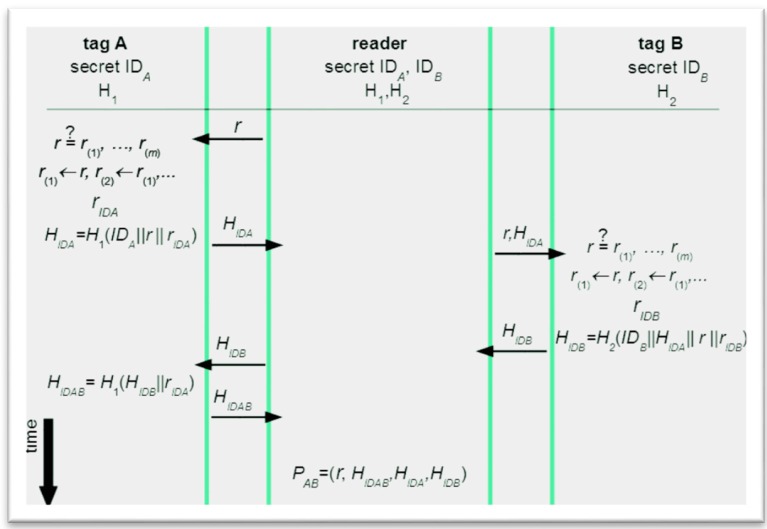
Yoking proof based grouping scheme enhanced by ND-PEPS.

The protocol given in Figure 4 can be relatively easily upgraded in such a way that not only Yoking proofs are enabled, but also privacy is achieved. This is done by applying the ND-PEPS principle and the obtained architecture is presented in Figure 5.

In the first step a challenge *r* is sent and checked by tag A for freshness. If *r* is fresh, it enters FIFO and the oldest *r* is discarded. Next, the tag calculates a random *r_IDA_* and concatenates it with its ID and *r*. The calculated *r_IDA_* is from a pre-defined interval, so upon receipt, the verifier generates all values from this interval using all tags’ IDs that are kept in its database, and obtains all possible hashed sequences. These sequences serve to find a match with the received part of *P*_AB_ to identify tag A. Similarly, using the rest of exchanged messages and taking into account their cascading, the verifier can analyze the proof *P*_AB_, while the identities of A and B remain hidden to an outside observer (and even to the reader, if it is not the verifier).

## 4. Discussion

The developed solution as this is the case with the original Yoking proofs is intended for off-line grouping proofs (as discussed earlier, on-line grouping proofs are better replaced by on-line authentication schemes where proofs of simultaneous reading can be formed by back-end systems). Further, in order to preserve its general applicability (without a need for RFIDs that use group keys, *etc.*) the improved protocol requires a minimal number of secrets to be stored on RFIDs in principle, only tags’ *ID*s. However, as also suggested in the above text (the attack on time-stamp with Peres-Lopez protocol), integrity provisioning of exchanged messages should be deployed. This would require an additional shared secret between a tag and a reader (or back-end system) and this secret would be used to derive hashed message integrity codes, MICs. Such MICs could be verified also by tags, not only readers (these checks are intentionally not included in Figure 5 to preserve clarity). Further, responses from readers are constantly changing in order to provide privacy through anonymity and untraceability—only authenticated readers (or back-end systems) will be able to find a match. Therefore, not only authentication of tags, but also (indirect) authentication of readers (or back-end systems) is achieved. Further, replies are prevented, because the exchanged cryptograms are logically linked to one session and to a particular group of participating RFIDs. As informal analyses are often subject to mistakes, the new protocol from Figure 5 has been thoroughly formally analyzed (see the appendix).

However, simplicity, clarity, and elegance of the protocol come at a price. First, invalid proofs cannot be prevented *per se*, although it is questionable if they can be achieved at all without on-line communications (these falsified proofs may go on the account of malfunctioning or rogue tags, or malicious readers). Next, the scheme is not providing an absolute time of when the tags group was scanned, but only a proof that such scanning has taken place at some time in the past (again, this seems to be an inherent problem for off-line grouping proofs). However, if the reader can be operated in a reliable manner (e.g., being a secure tablet operated by a trusted doctor with an access to a timing base), this functionality is provided. Further, replay attacks are prevented, as well as one-time proof is obtained to the degree enabled by that technological reality on RFID chips and, e.g., their pseudo-number generators.

Getting to computational costs—a large part of IoT devices lacks computing power and has the following typical structure: communications part, processing gates, memory gates and optionally one or more sensors. As communication is on RF such devices obviously need receiver and transmitter parts—these parts are, of course, excluded from calculation because they are needed regardless of an implemented a protocol. For the rest of the gates, computing resources are varying and these should be quantified.

One appropriate metric is described in [13]. It is based on technological reality of RFIDs production, where NAND gates are used. The total number of NAND gates required for a protocol gives the quantitative value about how lightweight a protocol is. This total is obtained by summing the gates needed for processing (*i.e.*, to implement protocol by using appropriate Boolean functions), the gates needed for storage (of identifiers, secret values and their calculations), and the gates that are equivalent to storage gates that would be needed to transfer bits, which are exchanged between a reader and a tag. Further, cryptographic protocols are built by using certain building blocks. Starting with memory cells, these are D cells and storing one bit with such cells requires five NAND gates [19]. To perform addition, eleven gates are needed for one bit full-adder, while addition modulo 2*^n^* requires *n*-times eleven NAND gates, and bitwise XOR requires four gates for each pair of bits [19]. Pseudorandom number generators can be implemented with shift registers and a shift register with four bits requires approximately 60 NAND gates, with approximately eight bits, 120 NAND gates, and so on [20] (using digital circuit artifacts quality random number generators can be built with approximately 300 NAND gates [21], while a more promising solution suited for EPCGen2 tags is given in [22]). Symmetric encryption can be done with light DES that requires approximately 1800 gates [23], while AES requires approximately 3000 gates [24]. Strong one-way hash functions are generally expensive—for example, SHA-3 can be implemented with approximately 2500 gates [25] (unfortunately, it seems SHA-3 has a built-in side channel [26]). Recent advancements are slowly making realistic also deployment of asymmetric encryption, so, for example, a 1024 bit public key scheme can be implemented with approximately 4600 logic gate equivalents [27]. Finally, as stated in [4], “For a protocol to qualify as being lightweight, the typical five-cent production cost limit has to be taken into account, which means that today one could count with 5000 to 8000 logic gates being dedicated to security and privacy”.

Let us analyze the part for tag A, which is more demanding than that for tag B. The tag has to store its ID and this requires 96 × 5 NAND gates. Further, it has to compute a strong one-way hash function by using DESL, with approximately 1800 logic gates needed. Further, a pseudo-random value is required, and assuming that its length is eight bits, 120 NAND gates are needed. To prevent exhaustive challenges attack and to ensure their freshness, 64 bits for challenges are assumed and twenty of them are stored in the FIFO, which results in 64 × 5 × 20 storage cells (to extend the range of stored challenges, only the first *k*-bits of each challenge can be stored in FIFO). The total number of gates for storage and processing is therefore approx. 2920 gates (the gates needed for comparing the new challenges with the stored ones have been neglected). Now the cost of communications has to be taken into account. Assuming that hashed outputs are also 64 bits long, the related cost is 64 × 5 × 4 NAND gates (four 64 bit-long messages are exchanged between tag A and a reader). Therefore the total estimated cost of the protocol is 4180 logic gates and the obtained solution is lightweight. Even when increasing its strength by extending its challenges and hashed values to 128 bits, this would keep its cost within the boundaries of lightweight protocols.

Now, how to relate the number of NAND gates to power consumption? A NAND gate in CMOS technology consists of four transistors. Each of them is subject to static and dynamic power consumption. The first part is bound by leakage current, while multiplying it by supply voltage gives static power consumption. Getting to dynamic power consumption, it consists of transient power consumption and capacitive-load power consumption. As to the transient power consumption (for a single bit switching), this is a product of a dynamic power-dissipation capacitance, square of supply voltage and input signal frequency [28]. As to the capacitive-load power consumption, this can be usually neglected compared to transient power consumption [28].

And finally, the properties of the new scheme are summarized in Table 1.

**Table 1:** Summary of security related properties of the new solution (*on-line mode depends on reader’s real-time access to a back end system).

Authentication	Privacy	Data-Base Desync Prevention	Tracking Prevention	On-Line Mode	Replay Prevention	Forge Proof	Lightweight
yes	yes	yes	yes	yes*	(yes)	yes	yes

## 5. Conclusions

Numerous areas of our lives are becoming increasingly dependent on IoT structures that range from NFCs, RFIDs, RFIDs with sensors, and bare sensors on one side of the spectrum to capable sensor (and actuator) motes’ networks on the other side of the spectrum. One common characteristic of these structures are stringent power (energy) requirements, while a large part of this digital ecosystem also lacks computing resources. Further, the need for grouping proofs for such structures is growing. One such example are proofs of simultaneous readouts from wireless medical sensors body area networks that are required together with privacy provisioning, and another is controlled drugs administration. To improve economic feasibility of such networks and to ensure a minimal interference with the patients’ activities, the deployed devices have to be small, which means that weak devices will be deployed, and that they will be passively powered. Therefore, lightweight grouping proofs with privacy provisioning for such structures are high on the agenda.

However, history is teaching us that the design of secure grouping proofs is an elusive endeavor. This paper, therefore, uses an approach where already-developed solutions (which have been subject to public scrutiny) are taken as a basis. Next, it improves their design to compensate the discovered weaknesses. As a result, a new grouping proof protocol is presented that is based on elegant Yoking proof scheme. By applying a so called non-deterministic principle it is further enhanced to provide privacy. Next, it is analyzed from security point of view, and from the computing requirements point of view. By using a tangible metric it is proved that the presented solution is lightweight, while the appendix contains a formal proof of its security.

Summing up, the number of IoT objects (like those in medical and assisted living settings) is expected to exceed other kinds of devices connected to the Internet. Therefore, the related security research area will be gaining importance. Clearly, as there is no size-fits-all solution, emerging areas of applications will call for new solutions like those described in [29], but grouping proofs can already be identified as being one of them.

## Appendix

This appendix gives a formal specification and verification of the scheme from Section 3.2. It is needed for verification with AVISPA model checkers, which use the widely used and most universal Dolev-Yao adversary model [30] (thus specific attacks like distance-bounding attack are excluded—for their deeper analysis a reader is referred to [31]). The specification is in HLPSL and the details about this language can be found in [32] (also without familiarity with HLPSL, its specifications can be mainly understood by readers who have some knowledge of formal verification techniques).

role role_A(A:agent,B:agent,R:agent,H1:hash_func,IDa:text,SND,RCV:channel(dy)) played_by A def= local State:nat,Ridb:text,Rida:text,IDb:text,Rand:text,H2:hash_func init State := 0 transition 1. State=0 /\ RCV(Rand') =|> State':=1 /\ Rida':=new() /\ secret(IDa',sec_3,{A,R}) /\ SND(H1(IDa.Rand'.Rida')) 5. State=1 /\ RCV(H2(IDb'.H1(IDa.Rand.Rida).Rand.Ridb')) =|> State':=2 /\ secret(IDa',sec_3,{A,R}) /\ secret(IDb',sec_4,{B,R}) /\ SND(H1(Rida.H2(IDb'.H1(IDa.Rand.Rida).Rand.Ridb'))) end role role role_B(A:agent,B:agent,R:agent,H2:hash_func,IDb:text,SND,RCV:channel(dy)) played_by B def= local State:nat,Ridb:text,Rida:text,IDa:text,H1:hash_func,Rand:text init State := 0 transition 3. State=0 /\ RCV(Rand'.H1(IDa'.Rand'.Rida')) =|> State':=1 /\ secret(IDa',sec_3,{A,R}) /\ Ridb':=new() /\ secret(IDb',sec_4,{B,R}) /\ SND(H2(IDb.H1(IDa'.Rand'.Rida').Rand'.Ridb')) end role role role_R(A:agent,B:agent,R:agent,H1:hash_func,H2:hash_func,IDa:text,IDb:text,SND,RCV:channel(dy)) played_by R def= local State:nat,Ridb:text,Rida:text,Rand:text init State := 0 transition 1. State=0 /\ RCV(start) =|> State':=1 /\ Rand':=new() /\ SND(Rand') 2. State=1 /\ RCV(H1(IDa.Rand.Rida')) =|> State':=2 /\ secret(IDa',sec_3,{A,R}) /\ SND(Rand.H1(IDa.Rand.Rida')) 4. State=2 /\ RCV(H2(IDb.H1(IDa.Rand.Rida).Rand.Ridb')) =|> State':=3 /\ secret(IDa',sec_3,{A,R}) /\ secret(IDb',sec_4,{B,R}) /\ SND(H2(IDb.H1(IDa.Rand.Rida).Rand.Ridb')) 6. State=3 /\ RCV(H1(Rida.H2(IDb.H1(IDa.Rand.Rida).Rand.Ridb))) =|> State':=4 /\ secret(IDa',sec_3,{A,R}) /\ secret(IDb',sec_4,{B,R}) end role role session1(A:agent,B:agent,R:agent,H1:hash_func,H2:hash_func,IDa:text,IDb:text) def= local SND3,RCV3,SND2,RCV2,SND1,RCV1:channel(dy) composition role_R(A,B,R,H1,H2,IDa,IDb,SND3,RCV3) /\ role_B(A,B,R,H2,IDb,SND2,RCV2) /\ role_A(A,B,R,H1,IDa,SND1,RCV1) end role role environment() def= const hash_0:function,ida:text,h1:hash_func,tagb:agent,taga:agent,reader:agent,h2:hash_func,idb:text,auth_1:protocol_id,auth_2:protocol_id,sec_3:protocol_id,sec_4:protocol_id intruder_knowledge = {reader,h1,h2} composition session1(taga,tagb,reader,h1,h2,ida,idb) end role goal authentication_on auth_1 authentication_on auth_2 secrecy_of sec_3 secrecy_of sec_4 end goal environment()
